# Antibacterial Activity and Anti-Quorum Sensing Mediated Phenotype in Response to Essential Oil from *Melaleuca bracteata* Leaves

**DOI:** 10.3390/ijms20225696

**Published:** 2019-11-14

**Authors:** Wenting Wang, Xiaoqin Huang, Huixiang Yang, Xianqian Niu, Dongxiang Li, Chao Yang, Liang Li, Liting Zou, Ziwen Qiu, Shaohua Wu, Yongyu Li

**Affiliations:** 1 College of Horticulture, Fujian Agriculture and Forestry University, Fuzhou 350002, China; 2160371001@fafu.edu.cn (W.W.); 1180371001@fafu.edu.cn (X.H.); 3180330047@fafu.edu.cn (H.Y.); lidongxiang@fafu.edu.cn (D.L.); 2160371002@fafu.edu.cn (C.Y.); 2160305002@fafu.edu.cn (L.L.); 1170371003@fafu.edu.cn (L.Z.); 1180371008@fafu.edu.cn (Z.Q.); 2 Fujian Institute of Tropical Crops, Zhangzhou 363001, China; nxq828@126.com

**Keywords:** *Melaleuca bracteata*, essential oil, gas chromatography-mass spectrometry (GC-MS), chemical components, antibacterial activity, pathogens, sub-minimal inhibitory concentrations (sub-MICs), quorum sensing (QS), bacterial contamination

## Abstract

The prominent antibacterial and quorum sensing (QS) inhibition activity of aromatic plants can be used as a novel intervention strategy for attenuating bacterial pathogenicity. In the present work, a total of 29 chemical components were identified in the essential oil (EO) of *Melaleuca bracteata* leaves by gas chromatography-mass spectrometry (GC-MS). The principal component was methyleugenol, followed by methyl trans-cinnamate, with relative contents of 90.46% and 4.25%, respectively. Meanwhile, the antibacterial activity and the QS inhibitory activity of *M. bracteata* EO were first evaluated here. Antibacterial activity assay and MIC detection against seven pathogens (*Dickeya dadantii* Onc5, *Staphylococcus aureus* ATCC25933, *Pseudomonas* spp., *Escherichia coli* ATCC25922, *Serratia marcescens* MG1, *Pseudomonas aeruginosa* PAO1 and *Chromobacterium violaceum* ATCC31532) demonstrated that *S. aureus* ATCC25933 and *S. marcescens* MG1 had the higher sensitivity to *M. bracteata* EO, while *P. aeruginosa* PAO1 displayed the strongest resistance to *M. bracteata* EO. An anti-QS (anti-quorum sensing) assay revealed that at sub-minimal inhibitory concentrations (sub-MICs), *M. bracteata* EO strongly interfered with the phenotype, including violacein production, biofilm biomass, and swarming motility, as well as *N*-hexanoyl-L-homoserine lactone (C6-HSL) production (i.e., a signaling molecule in *C. violaceum* ATCC31532) of *C. violaceum*. Detection of C6-HSL indicated that *M. bracteata* EO was capable of not only inhibiting C6-HSL production in *C. violaceum*, but also degrading the C6-HSL. Importantly, changes of exogenous C6-HSL production in *C. violaceum* CV026 revealed a possible interaction between *M. bracteata* EO and a regulatory protein (cviR). Additionally, quantitative real-time polymerase chain reaction (RT-qPCR) analysis demonstrated that the expression of QS-related genes (*cviI*, *cviR*, *vioABCDE*, *hmsNR*, *lasA-B*, *pilE1*, *pilE3*, and *hcnB*) was significantly suppressed. Conclusively, these results indicated that *M. bracteata* EO can act as a potential antibacterial agent and QS inhibitor (QSI) against pathogens, preventing and controlling bacterial contamination.

## 1. Introduction

It is well documented that the large-scale use of chemical antimicrobials and antibiotics causes resistance in pathogenic microorganisms. Bacteria rapidly mutate and adapt in response to new hostile environments [1]. Therefore, it is not a wise solution that the use of these antimicrobials be kept to a minimum or until pathogens are halted. Recently, natural antimicrobials from aromatic plants have attracted attention as alternatives to chemical ones [2]. Essential oils (EOs), the secondary metabolites of aromatic plants, are used to prevent bacterial infections due to their prominent antibacterial activity and quorum sensing (QS) inhibition. They are also safe and nontoxic compounds, meeting the requirements for green antibacterial agents [3]. 

QS is a cell-density-dependent mechanism used by bacteria to regulate gene expression [4]. Bacteria release autoinducers (AIs) or signals that realize cell-to-cell communication, and AIs have been identified as oligopeptides and *N*-acyl-homoserine lactones (AHLs) in Gram-positive and Gram-negative bacteria, respectively [5]. Numerous studies have shown that bacteria rely on QS systems to orchestrate the synchronous secretion of virulence factors (VFs) and biofilm formation [6]. Potential QS inhibitors (QSIs) can reduce the bacterial pathogenicity and target bacterial QS systems rather than killing cells, which can reduce or slow the selective pressure for developing resistance [7,8]. *Chromobacterium violaceum* ATCC31532, a well-documented Gram-negative bacterium, has been used widely in screening QS inhibitors and in researching the QS inhibitory mechanism, due to its visible violacein [9] and clear QS regulatory system. *N*-hexanoyl-l-homoserine lactone (C6-HSL), which is modulated by the *cviI* gene, binds to the transcriptional regulator to regulate biofilm formation, swarming movement, and the secretion of virulence factors such as violacein and exopolysaccharide (EPS) [10]. It was previously found that sub-minimal inhibitory concentration (sub-MIC) EO levels of green cardamom, rose, clove, and chamomile are capable of blocking the network in *C. violaceum* [11,12]. In this light, plant EOs are expected to be emerging QSIs to attenuate the virulence of pathogens and control bacterial infections and drug resistance.

*Melaleuca* is a genus of plants in the Myrtle family Myrtaceae, mainly in Australia, and several species have been introduced and cultivated in China [13]. Species from this genus are known to be good sources of antibacterial agents and medicinal materials. For example, “tea tree oil” derived from *M. alternifolia* is used in food processing to extend product shelf life [14]. *Melaleuca bracteata* is popularly exploited as an ornamental plant and is well known for its aromatic properties, as well as its vast medicinal properties. It is used to treat heart attack, stroke, infected wounds, skin disorders, and fungal infection. Furthermore, *M. bracteata* is also used to aid in stimulating glandular secretions and to reduce congestion in the veins, and its leaves constitute a component of an anti-HIV concoction. In addition, the stem bark extract of *M. bracteata* possesses antisecretory and antiulcerogenic activities [15]. The antibacterial and antioxidant activity of *M. bracteata* EO has recently been reported [16,17], but its anti-QS ability has never been described. Thus, we tested its antibacterial ability against pathogenic bacteria and anti-QS activity against *C. violaceum* ATCC31532 in this work, providing a theoretical basis for the development of *M. bracteata* EO as an antibacterial agent and QS inhibitor to prevent and control bacterial contamination.

## 2. Results

### 2.1. Analysis of the Components in *M. bracteata* EO by GC-MS

**Figure 1:**
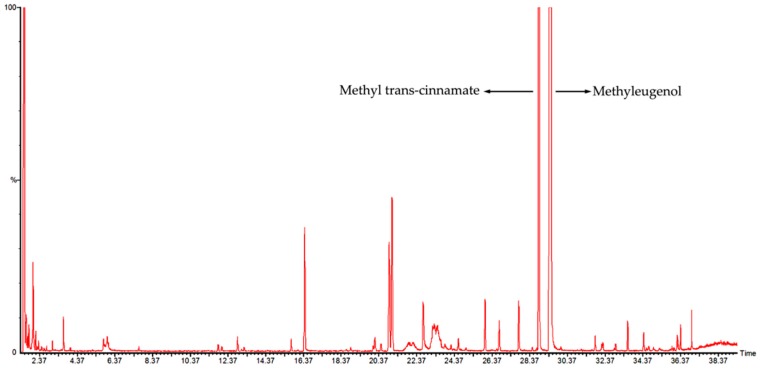
The GC-MS total ion chromatogram of *Melaleuca bracteata* essential oil (EO).

A total ion flow chromatogram of the *M. bracteata* EO analyzed by GC-MS is shown in Figure 1. The correlations of the peak area normalization method with the mass spectrometry database were determined to qualitatively and quantitatively analyze the components of the EO. Table 1 shows that 29 components were identified from the *M. bracteata* EO, accounting for 96.49% of the total contents, among which methyleugenol displayed the largest proportion, up to 90.46%, followed by methyl trans-cinnamate (relative content of 4.25%), and the relative content of other components was less than 1%.

### 2.2. Determination of the Antimicrobial Activity and MIC of *M. bracteata* EO

The antimicrobial activities of the *M. bracteata* EO were tested against the common pathogens *D. dadantii* Onc5, *S. aureus* ATCC25933, *Pseudomonas* spp., *E. coli* ATCC25922, *S. marcescens* MG1, *P. aeruginosa* PAO1, and *C. violaceum* ATCC31532 using the plate perforation method. *M. bracteata* EO exhibited an effective concentration-dependent inhibitory effect against all tested bacteria. *M. bracteata* EO showed stronger inhibition against *S. aureus* ATCC25933 and *S. marcescens* MG1 with higher inhibition ability (15.28 ± 1.083 mm and 14.11 ± 0.789 mm, respectively) than other test bacteria at the concentration of 80‰. *M. bracteata* EO exhibited antibacterial activity against *D. dadantii* Onc5, *P. aeruginosa* PAO1, *E. coli* ATCC25922, *Pseudomonas* spp., and *C. violaceum* ATCC31532 with inhibition zone diameters of 11.89 ± 0.246 mm, 10.47 ± 0.186 mm, 11.38 ± 0.286 mm, 13.42 ± 0.715 mm, and 11.55 ± 0.34 mm, respectively (Table 2).

The MIC of *M. bracteata* EO was assessed for all test pathogens using the double dilution method with concentrations varying from 80‰ to 0.625‰. The MIC of *M. bracteata* EO was 2.5‰ for *S. aureus* ATCC25933 and *S. marcescens* MG1, 5‰ for *Pseudomonas* spp., 10‰ for *D. dadantii* Onc5, *E. coli* ATCC25922, and *C. violaceum* ATCC31532, and 20‰ for *P. aeruginosa* PAO1 (Table 2).

Collectively, the results indicated that *S. aureus* ATCC25933 and *S. marcescens* MG1 had higher sensitivity to *M. bracteata* EO, while *P. aeruginosa* PAO1 showed the strongest resistance to *M. bracteata* EO.

### 2.3. Quorum Sensing Inhibition (QSI) assays of *M. bracteata* EO

**Figure 2:**
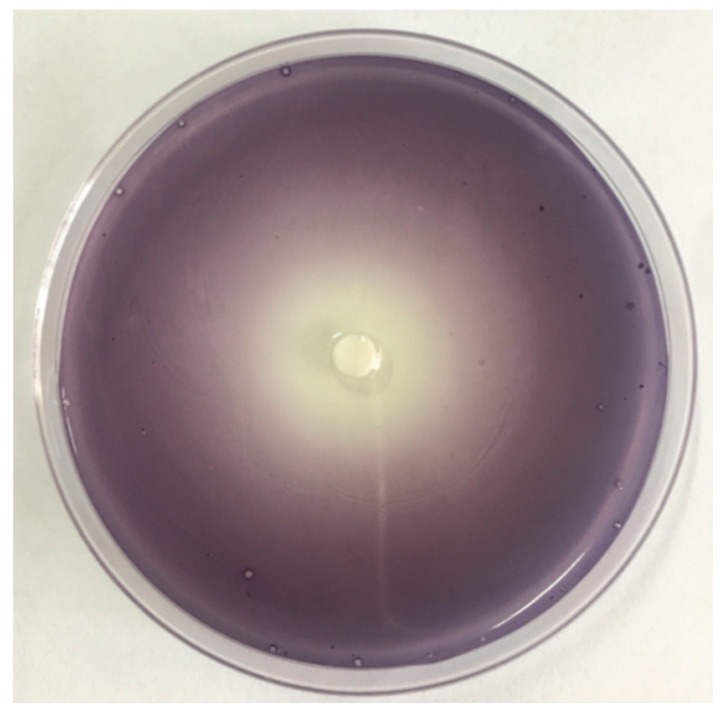
Quorum sensing inhibition (QSI) effect of *M. bracteata* EO on biosensor CV026.

The zone of non-purple pigment on agar plates and the extent of the inhibition of purple pigment in *C. violaceum* CV026 by *M. bracteata* EO were observed as shown in Figure 2, indicating that *M. bracteata* EO showed good inhibition for the QS-mediated violacein production of CV026. Hence, in the present study, *M. bracteata* EO at sub-MICs (5‰, 2.5‰, 1.25‰, and 0.625‰) was used for the further experiments.

### 2.4. Growth Curve

**Figure 3:**
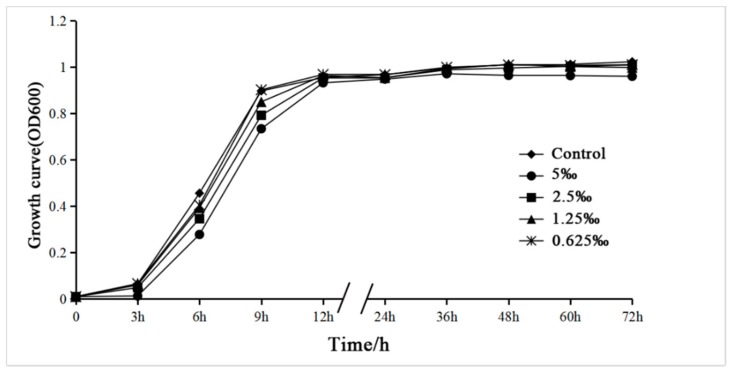
Effect of *M. bracteata* EO on the growth of *Chromobacterium violaceum*. Growth curves of *C. violaceum* treated with varying concentrations of EO: 5‰, 2.5‰, 1.25‰, and 0.625‰ (the control had no *M. bracteata* EO).

To confirm the non-antibacterial activity of *M. bracteata* EO at sub-MICs (5‰, 2.5‰, 1.25‰, and 0.625‰), the activities of *C. violaceum* treated with or without different concentrations of *M. bracteata* EO were determined for 0–72 h. The growth curve showed that *C. violaceum* treated with *M. bracteata* EO at sub-MICs began to enter the logarithmic phase later than the control. Nevertheless, the growth of *C. violaceum* did not differ between the control and treated groups in the stationary phase (>12 h) (Figure 3). These results revealed that *M. bracteata* EO was inefficient at inhibiting growth under the test conditions. We therefore assessed the specific effect of *M. bracteata* EO at sub-MICs (5‰, 2.5‰, 1.25‰, and 0.625‰) on QS in *C. violaceum*.

### 2.5. Determination of Violacein

**Figure 4:**
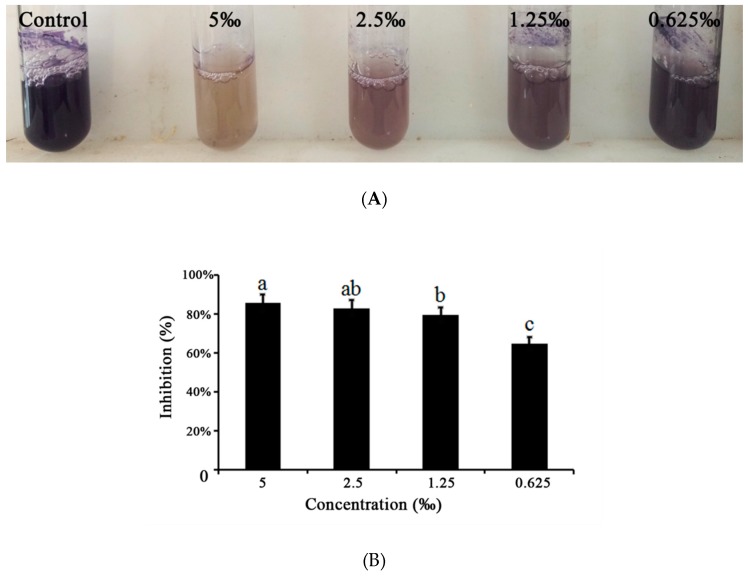
(**A**) Effect of *M. bracteata* EO on violacein in *C. violaceum*. (**B**) Quantitative analysis of violacein inhibition in *C. violaceum* by EO (5‰, 2.5‰, 1.25‰, and 0.625‰). Mean values of triplicate independent experiments and SD are shown. Bars indicate standard errors and different letters (a–c) above the bars represent significant differences (*p* < 0.05).

Violacein is the important metabolite of *C. violaceum* which is regulated by the QS system [18]. Therefore, we detected the effect of *M. bracteata* EO on the violacein in *C. violaceum*. In the quantitative assay, violacein inhibition reached a maximum of 85.47% in *C. violaceum* when treated with *M. bracteata* EO at 5‰ (the highest tested concentration) (Figure 4).

### 2.6. Biofilm Determination

**Figure 5:**
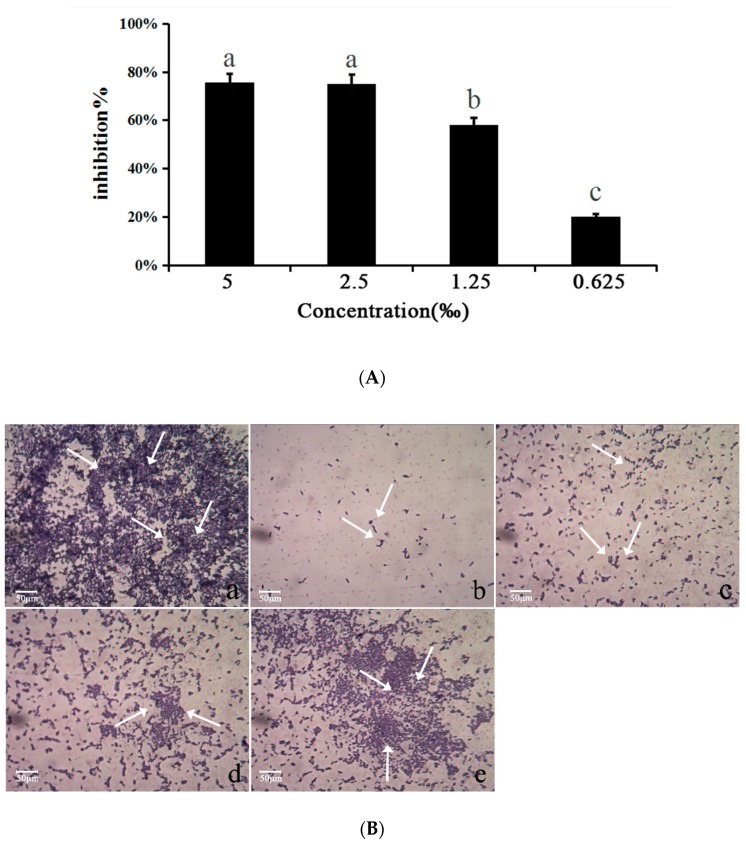
*M. bracteata* EO reduced biofilm formation of *C. violaceum*. (**A**) Quantitative assessment of biofilm biomass inhibition. Mean values of eight independent experiments and SD are shown. Bars indicate standard errors and different letters (a–c) above the bars represent significant differences (*p* < 0.05). (**B**) Light microscope images (a–e) under a light microscope at a magnification of 40×. a: untreated; b: 5‰; c: 2.5‰; d: 1.25‰; e: 0.625‰. The arrows indicate the dyed biofilm.

The quantitative biofilm assay demonstrated that treatment with *M. bracteata* EO (5‰, 2.5‰, 1.25‰, and 0.625‰) inhibited the biofilm biomass of *C. violaceum* in a concentration-dependent manner, and the inhibition of biofilm biomass were 75.56%, 75.03%, 58.16%, and 20.2%, respectively (Figure 5A). In addition, *M. bracteata* EO was found to be very effective in inhibiting biofilm formation of *C. violaceum* based on observations by light microscope (Figure 5B).

### 2.7. Swarming Motility Assay

**Figure 6:**
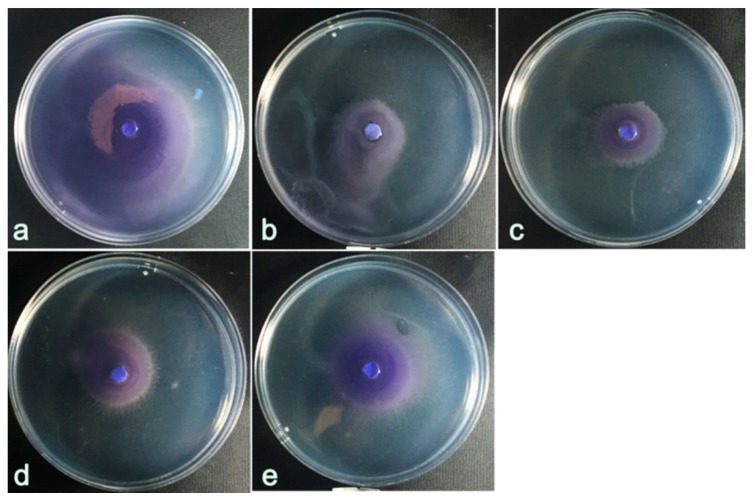
Effect of *M. bracteata* EO at different concentrations (5‰, 2.5‰, 1.25‰ and 0.625‰) on the swarming motility of *C. violaceum*. **a**: control, untreated with EO. **b**–**e**: treated with EO concentrations of 5‰, 2.5‰, 1.25‰, and 0.625‰.

Swarming migration plays an important role in QS-regulated biofilm formation in pathogens [19,20]. An effort was made to examine the anti-QS potential of *M. bracteata* EO against swarming motility in *C. violaceum*. The results showed that *M. bracteata* EO disturbed the swarming behavior of *C. violaceum*, and the maximum inhibition was recorded at the highest concentration (5‰) (Figure 6).

### 2.8. Detection of the Production of C6-HSL Signal Molecules

The C6-HSL production was measured through a well-diffusion assay using CVO26 as the monitor strain, and the concentration of C6-HSL extracts was estimated by measuring the diameter of the violacein induced zone. The extracts from *C. violaceum* treated with or without *M. bracteata* EO (5‰, 2.5‰, 1.25‰, and 0.625‰) suggested that *M. bracteata* EO is able to reduce the C6-HSL production in a concentration-dependent manner (Figure S1).

**Figure 7:**
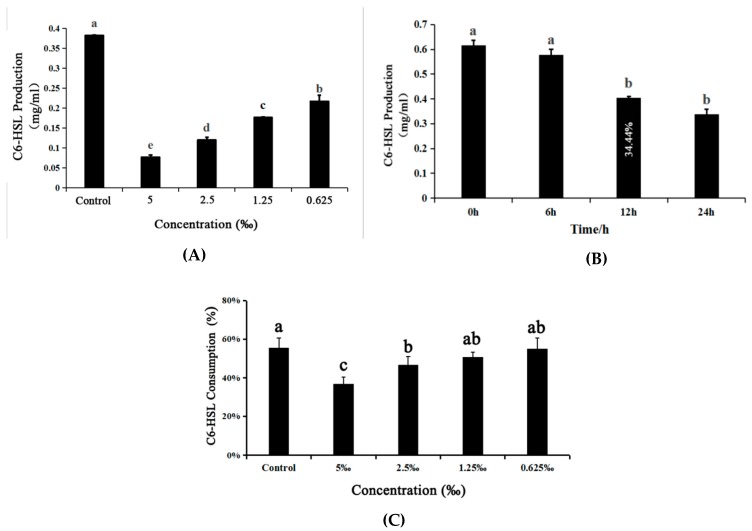
(**A**) Effect of *M. bracteata* EO at different concentrations (5‰, 2.5‰, 1.25‰ and 0.625‰) on C6-HSL of *C. violaceum*. (**B**) Effect of *M. bracteata* EO (5‰) on C6-HSL treated for 0, 6, 12, and 24 h. The degradation of *N*-hexanoyl-l-homoserine lactone (C6-HSL) was 34.44% at 12 h. (**C**) Effect of *M. bracteata* EO at different concentrations (5‰, 2.5‰, 1.25‰, and 0.625‰) on C6-HSL of *C. violaceum* CV026. The results are the mean (*n* = 3) ± standard deviation. Bars indicate standard errors and different letters (a–c) above the bars represent significant differences (*p* < 0.05).

To verify the biosensor screening results, the C6-HSL extracts were analyzed quantitatively by GC technology. According to the retention time and the standard curve, the concentration of C6-HSL in the extracted samples was calculated (Figure S2). In agreement with observations in the CV026 biosensor, the concentration of C6-HSL in the treated groups decreased significantly compared with the control. In addition, the concentrations in the control group and the treatment group (5‰, 2.5‰, 1.25‰, and 0.625‰ *M. bracteata* EO) were 0.38, 0.08, 0.12, 0.18, and 0.22 mg/mL, respectively (Figure 7A and Figure S3). Our data confirmed that *M. bracteata* EO was able to repress C6-HSL production in *C. violaceum*, which resulted in the attenuation of bacterial virulence.

The capacity of *M. bracteata* EO to target C6-HSL directly was determined by adding exogenous C6-HSL to LB broth supplemented with *M. bracteata* EO extracts at the highest tested concentration of 5‰. Compared to the control, the extract had no significant effect at 6 h, and then a significant drop in the C6-HSL content was observed with the increase in incubation time (at 12 h and 24 h) (Figure 7B and Figure S4). These data suggest that *M. bracteata* EO can directly degrade C6-HSL.

In addition, the C6-HSL extracts from *C. violaceum* CV026 treated with *M. bracteata* EO (5‰, 2.5‰, 1.25‰, and 0.625‰) showed that the consumption of exogenous C6-HSL with *M. bracteata* EO treatment was lower than that of the control. Among the exogenous C6-HSL with the lowest consumption was at the highest tested concentration of 5‰, independent of a direct effect on growth (Figure 7C and Figure S5). The results indicate that *M. bracteata* EO may be able to interact with the cviR protein.

Collectively, we speculated that *M. bracteata* EO not only degraded the C6-HSL directly and inhibited C6-HSL production, but also interacted with the cviR protein.

### 2.9. *M. bracteata* EO Reduced the Expression of the QS-Related Genes

**Figure 8:**
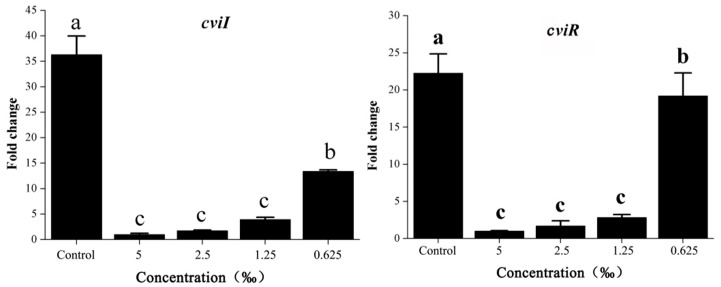
Effect of *M. bracteata* EO on the expression of *cviI* and *cviR*. Expression of the house-keeping gene *rpoD* was used as the internal control for each sample. Bars indicate standard errors and different letters (a–c) above the bars represent significant differences (*p* < 0.05).

**Figure 9:**
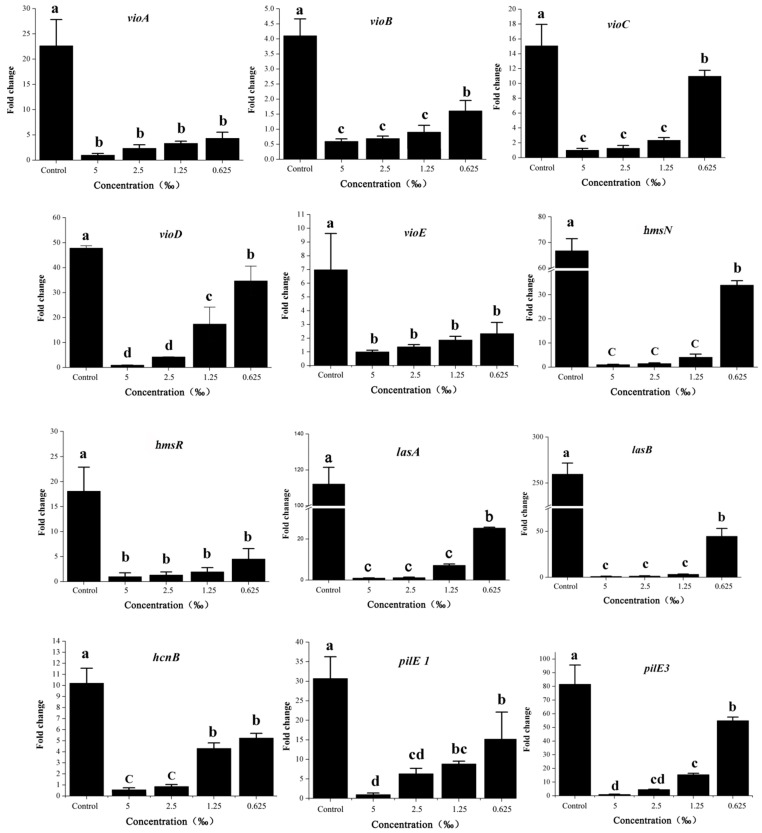
Effect of *M. bracteata EO* on the expression of genes regulated by LuxI–LuxR system. *vioA, vioB, vioC, vioD, vioE, hmsN, hmsR, lasA, lasB, hcnB, pilE1*, and *pilE3* were detected in response to *M. bracteata* EO treatment. Expression of the house-keeping gene rpoD was used as the internal control for each sample. The *M. bracteata* EO treatment concentrations were 5‰, 2.5‰, 1.25‰, and 0.625‰. Control was untreated. Bars indicate standard errors, and different letters (a–c) above the bars represent significant differences (*p* < 0.05).

*cviI*/*cviR* are important regulatory factors in the QS system of *C. violaceum* [18]. Accordingly, we assessed the effects of *M. bracteata* EO on the main regulatory factors *cviI* and *cviR* in *C. violaceum* ATCC 31532 by RT-qPCR. As expected, the expression of *cviI* and *cviR* revealed a dose-dependent downregulation in response to *M. bracteata* EO (5‰, 2.5‰, 1.25‰, and 0.625‰) in comparison with the control (Figure 8). Furthermore, we detected the effect of methyleugenol (ME) on the expression of important virulence factors, including violacein production (via *vioA–E*), biofilm formation (via *hmsNRHF*), and elastase production (via *lasA* and *lasB*) [21]. These genes are downstream of the QS cascade and controlled by the QS system. In the presence of *M. bracteata* EO, the expression of *vioA, vioB, vioC, vioD, vioE, hmsN, hmsR, lasA*, and *lasB* were clearly suppressed relative to the control group (Figure 9). However, the expression of *hmsH* and *hmsF* was not affected by *M. bracteata* EO (Figure S6).

Studies have revealed that pilus (*pilE1–3*) and cyanide production (*hcnA–C*) were associated with the QS system in *C. violaceum* [22]. Considering our observation that the *cviI*/*cviR* system was significantly suppressed in response to EO, we decided to monitor the change in *pilE1*–3 and *hcnA*–*C*. Although other genes were significantly repressed by *M. bracteata* EO, *hcnA*, *hcnC,* and *pilE2* were not (Figure 9 and Figure S6). It is possible that these genes were not directly or independently controlled by the QS system. Collectively, our data suggest that *M. bracteata* EO can be used to inhibit the expression of key virulence genes of *C. violaceum*, independent of a direct effect on growth rate.

## 3. Discussion

A variety of aromatic plants have been reported to have antibacterial activities and anti-quorum sensing activities [7,23]. *M. bracteata*, as an excellent medicinal aromatic plant with colorful leaves, has great development and utilization value. In this paper, we evaluated the antimicrobial activity of *M. bracteata* EO against seven pathogens, and its potential anti-QS activity was detected for the first time with *C. violaceum* ATCC31532.

*M. bracteata* EO was capable of inhibiting pathogens, including: *D. dadantii* Onc5, *S. aureus* ATCC25933, *Pseudomonas* spp., *E. coli* ATCC25922, *S. marcescens* MG1, *P. aeruginosa* PAO1, and *C. violaceum* ATCC31532. Among them, *D. dadantii* Onc5 and *Pseudomonas* spp. are pathogenic bacteria that cause plant soft rot. The results showed that *M. bracteata* EO had stronger inhibition against *S. aureus* ATCC25933 and *S. marcescens* MG1, and *P. aeruginosa* PAO1 was generally more resistant than the other organisms tested in this paper, which were well-matched with the MIC results. A study by Siddique et al. [16] showed that *M. bracteata* EO exhibited antimicrobial activities against pathogens including Gram-positive (*Bacillus subtilis* subsp *spizizenii*, *Staphylococcus aureus*) and Gram-negative bacteria (*Enterobacter aerogenes*, *Escherichia coli*, *Klebsiella pneumonia*, *Pseudomonas aeruginosa,* and *Salmonella enterica*). Collectively, *M. bracteata* EO has the potential to act as a natural antibacterial agent due to its outstanding and broad-spectrum antimicrobial activity. Some antimicrobial agents with broad-spectrum antimicrobial activity have QS inhibitory activities under sub-MICs [23,24]. Thus, we are very interested in whether *M. bracteata* EO is capable of suppressing bacterial pathogenicity based on interfering with QS systems instead of having a direct effect on growth.

In the present study, we have systematically studied the inhibitory effect of *M. bracteata* EO (sub-MICs) on the QS system of *C. violaceum* from the QS phenotype and at the molecular level. We detected the QS inhibitory potential of *M. bracteata* EO based on its ability to inhibit the production of AHL-dependent virulence factors such as violacein in *C. violaceum*. It has previously been confirmed that the compound, which has the ability to inhibit violacein production without influencing the growth of *C. violaceum*, is considered to be a promising QS inhibitor [25,26]. Biofilm formation plays an important role in bacterial pathogenicity and decreases drug sensitivity. Thus, interfering with biofilm formation might be a preferable and convenient way to attenuate the virulence of disease pathogens as well as drug resistance [27,28]. A biofilm detection assay showed that *M. bracteata* EO not only reduced the biofilm biomass but also influenced the colony formation, as evidenced by the light microscope images in Figure 5B. The inhibition response of natural products as found in our study is also supported by the findings on EO of *Cuminum cyminum* [29] and *Mentha piperita* [30], which also influenced the biofilm biomass and formation of *Pseudomonas aeruginosa*, *C. violaceum,* and *Aeromonas hydrophila*. Swarming involves the differentiation of vegetative cells into hyper-flagellated swarm cells that undergo rapid and coordinated population migration across solid surfaces, and swimming and swarming behavior essentially determine the biofilm formation [20]. Thus, the inhibition of swarming motility by *M. bracteata* EO partially accounted for the reduction in the biofilm biomass and disruption of biofilm architecture in *C. violaceum*. We next employed RT-qPCR to evaluate the expression of QS-regulated genes (*vioA-E*, *hmsNRHF*) in *C. violaceum*. The RT-qPCR results did not correlate well with biofilm results. The expression of *hmsHF* did not decrease in response to *M. bracteata* EO in a concentration-dependent manner compared with that of control. The above data indicate that biofilm formation is a sophisticated process and that QS is a vital regulatory mechanism for biofilm formation, but not the only one.

According to the working model of QS regulation, the LuxR receptors are unstable, and rapidly degraded at low AI concentrations. As the cell density increases, the accumulated AI binds the LuxR-type receptor, leading to stabilization of the LuxR–AI complex, which subsequently binds DNA in promoters, driving genes regulated by QS [31]. Thus, QS quenching can be achieved either by interruption of AI signal generation, inhibition of AI signal dissemination, or inhibition of AHL signal reception [32]. The reduction in C6-HSL concentrations in *C. violaceum* culture treated with *M. bracteata* EO could be the result of either (1) inhibition of cell growth, (2) degradation of C6-HSL, or (3) inhibition of the C6-HSL production. With regard to the first hypothesis, it was observed that the growth tendencies of *C. violaceum* treated and untreated with *M. bracteata* EO were similar, without obvious differences after 12 h. The second hypothesis refers to the potential degradation of C6-HSL by *M. bracteata* EO. The quantification of C6-HSL in inoculated broth treated with *M. bracteata* EO (the highest tested concentration of 5‰) revealed that *M. bracteata* EO was able to directly degrade C6-HSL (Figure 7B). Regarding the third hypothesis, a dose-dependent reduction of C6-HSL production in *C. violaceum* showed that *M. bracteata* EO interfered with the accumulation of C6-HSL (Figure 7A, Figure S2). When it is taken into account that C6-HSL degradation was approximately 34.44% in the presence of 5‰ *M. bracteata* EO for 12 h (Figure 7B), the inhibition of QS by *M. bracteata* EO could attributed to its capacity to inhibit C6-HSL production.

Moreover, the antagonists targeting the LuxR receptors also have huge potential for anti-QS development. Chloro thiolactone (CTL) and chloro lactone (CL) were shown to be cviR antagonists that prevent cviR from binding the promoter DNA of regulated genes [33]. Interestingly, we found that the consumption of exogenous C6-HSL was lowest in CV026 upon treatment with *M. bracteata* EO (at the highest tested concentration of 5‰); we inferred that *M. bracteata* EO can interact with cviR. Collectively, the findings in this work strongly support that *M. bracteata* EO acts through the QS machinery to inhibit specific virulence determinants in *C. violaceum*. The possibility of QS suppression by *M. bracteata* EO consists mainly of interactions with cviR and/or cviI proteins, directly resulting in the suppression of C6-HSL production, and is partially related to the degradation of C6-HSL production.

This finding is consistent with previous studies [34] which indicated that methyleugenol (ME) was the major constituent of *M. bracteata* EO, with a relative content of up to 90.46%. A study by Packiavathy et al. showed that ME exhibited violacein inhibition without growth inhibition [29]. Consistent with the above findings, ME also exhibited QSI activity toward the biosensor CV026 in the present study (Figure S7). Thus, we inferred that ME may play a leading role in the suppression of the QS system by *M. bracteata* EO. However, more work is clearly required in order to ascertain the exact binding site and molecular mechanism of action.

## 4. Materials and Methods

### 4.1. Essential Oil, Bacterial Strains, Medium, and Growth Conditions

The leaves of *M. bracteata* were collected from Fujian Agriculture and Forestry University (Fujian, China). Essential oil (EO) was extracted by steam distillation and then stored at −20 °C.

The microorganisms researched in this study were *Dickeya dadantii* Onc5, *Staphylococcus aureus* ATCC25933, *Pseudomonas* spp. (isolated from *Capsicum annuum* L), *Escherichia coli* ATCC25922, *Serratia marcescens* MG1, *Pseudomonas aeruginosa* PAO1, *Chromobacterium violaceum* ATCC31532, and *Chromobacterium violaceum* CV026, which were stocked in our laboratory. All tested bacteria were incubated in LB broth and grown under conditions of 30 °C, 150 rpm, and 12 h. The swarming motility medium consisted of 1% tryptone, 0.5% NaCl, 0.5% agar, and 0.5% d-glucose. *N*-Hexanoyl-l-homoserine lactone (C6-HSL) was purchased from Sigma-Aldrich (Shanghai, China). *n*-Alkanes (C8–C20) standard solution was purchased from Fluka (Shanghai, China).

### 4.2. Determination of Components of M. bracteata EO by GC-MS

The *M. bracteata* EO was subjected to GC-MS analysis using a GC (Clarus^®^680) equipped with a mass-selective detector (SQ8T) in electronic ionization (EI) mode and Turbomass Ver 6.1.0 software (Perkin Elmer Company, MA, America). Sample injection was performed in split mode (20:1) into a DB-5MS capillary column (30 m × 25 mm × 0.25 µm). Helium was used as the carrier gas at 1 mL/min. The GC injector temperature was set at 250 °C. The oven temperature program was optimized to hold at 50 °C for 2 min), finally increasing by 50 °C/min up to 250 °C (maintained for 2 min). The transfer line temperature was adjusted to 250 °C. Mass spectrometry conditions were as follows: electron ionization source set to 70 eV, MS transmission line to 250 °C, and MS source to 230 °C. The mass spectrometer was run in full-scan mode (*m/z* 45–550). The essential oil production was analyzed by peak area.

The essential compounds were identified on the basis of a comparison of their retention index (RI) relative to *n*-alkanes (C8–C20), standard substance, as well as published data and EI mass spectra from the literature. The relative mass fraction of the *M. bracteata* EO was calculated using the peak area normalization method.

The formula for the retention index (RI) is shown in Equation (1) [35,36]:

RI = 100Z + 100[RT(x) − RT(z)]/[RT(Z + 1) − RT(z)](1)

where Z is the number of carbons© in the smaller alkane; RT(x) is the retention time of the unknown compound; RT(z) is the retention time of the smaller alkane; and RT(Z + 1) is the retention time of the larger alkane.

### 4.3. Determination of Antimicrobial Activity and MIC of *M. bracteata* EO

The test strains, *D. dadantii* Onc5, *S. aureus* ATCC25933, *Pseudomonas* spp., *E. coli* ATCC25922, *S. marcescens* MG1, *P. aeruginosa* PAO1, and *C. violaceum*, were cultured in LB broth at 150 rpm and 30 °C for 12 h. All of the tested strains were adjusted to a microbial suspension of 10^9^ CFU/mL with distilled water. The *M. bracteata* EO was serially diluted to 80‰, 40‰, 20‰, and 10‰ with methanol. The plate perforation method was performed using the following procedure with some modifications [37]. Briefly, 1% test bacterial suspension (10^9^ CFU/mL) was added to the heated LB medium (containing 2% agar) at a temperature of 50 °C. After blending, the mixture was quickly poured into the Petri dishes. Then, 6-mm holes were punched in the solidified LB medium in the Petri dishes. A 35-µL volume of *M. bracteata* EO at different concentrations (80‰, 40‰, 20‰, and 10‰) was added to the holes. The culture plates were incubated at 30 °C. The antimicrobial activity was determined by measuring the antimicrobial diameters. Methanol and kanamycin (250 µg/mL) solutions was used as the negative control and positive control, respectively.

Double dilution method was applied to test the minimum inhibitory concentrations (MICs) of *M. bracteata* EO. The assay was performed using 1.5-mL microcentrifuge tubes and consisted of a gradient of *M. bracteata* EO—80‰, 40‰, 20‰, 10‰, 5‰, 2.5‰, 1.25‰, and 0.625‰—with the same final volume (150 μL), and 150 μL of bacterial suspension (1% of tested bacteria (OD600, 0.9) were added to LB medium). After mixing, the tubes were incubated at 30 °C and 150 rpm for 24 h and the OD600 was measured. Each assay was performed in triplicate.

### 4.4. Quorum Sensing Inhibition Assays

The QSI assay was performed on agar plates employing the biosensor strain *C. violaceum* CV026, which produces a purple pigment only in response to added exogenous AHLs [38].

The quorum sensing inhibition assay was performed according to the procedure described by Zhang et al. [39], with some modifications. An overnight culture of 1% CV026 was spread on LB plates (20 mL), and then exogenous C6-HSL solution was added to the plates. Filter paper (6 mm in diameter) was then placed on the center of the plate. Next, 15 µL of the *M. bracteata* EO and methyleugenol (ME) was added to filter paper, and the plates were incubated for 24 h at 30 °C. QSI was assessed from the formation of a ring of inhibition, as a result of violacein production, around the filter paper.

### 4.5. Growth Curve Analysis

The 1% *C. violaceum* (OD600, 0.9) was incubated in a 250 mL Erlenmeyer flask containing 20 mL of LB broth supplemented with *M. bracteata* EO (sub-MICs, 6% *v*/*v*), and the culture was mixed at 30 °C with shaking at 150 rpm in a rotatory shaker. The growth of bacteria was determined by UV–Visible spectrophotometry (U-290, Hitachi Company, Tokyo, Japan ) at OD600 from 0 to 72 h.

### 4.6. Violacein Detection Assay

Overnight culture of 1% *C. violaceum* (OD600, 0.9) was added into a glass tube containing 5 mL LB broth supplemented with *M. bracteata* EO (sub-MICs, 6% *v*/*v*), and the liquid was mixed and incubated at 30 °C for 12 h. The specific methods were as follows: 1 mL of the cultured solution was centrifuged in a 1.5 mL centrifuge tube at 4 °C and 12,000× *g* for 20 min; the bacteria and violacein were collected, and 1 mL of dimethyl sulfoxide (DMSO) was added to the centrifuge tube. The mixed liquid was vortexed at room temperature and centrifuged again for 3 min to obtain a purple-colored solution, and the violacein inhibition was measured by UV–Visible spectrophotometry at OD595. Each assay was performed in triplicate.

### 4.7. Effect of Essential Oil on Biofilm Development

The effect of *M. bracteata* EO on biofilm was assessed by staining and quantifying the biofilm biomass using a microtiter dish with crystal violet (CV), as previously described [40]. The biofilm biomass was quantified by measuring the absorbance at 550 nm in a microplate reader and recording the absorbance of CV dye bound to the biofilm. Each assay was performed in eight replicates.

To determine the ability of *M. bracteata* EO to disrupt the biofilm, a biofilm disruption assay was performed by following the revised method described previously [27]. Briefly, the test pathogens treated with different concentrations of EO (sub-MICs) were developed in six-well plates with cover glasses 1 cm × 1cm for 12 h, and the biofilms were stained with crystal violet. Then, the biofilm was observed under a light microscope (Bimuyiqi Company, Shanghai, China).

### 4.8. Swarming Motility

An effort was made to examine the effects of *M. bracteata* EO on the swarming motility in *C. violaceum*. The specific method is described briefly as follows [27]. First, 5 μL of overnight bacterial cultured bacterium (OD600, 0.9) was placed on 6-mm filter paper at the center of a plate containing swarming motility medium supplemented with *M. bracteata* EO (sub-MICs, 6% *v*/*v*) and incubated. The ability of swarming motility was measured by the migration distance.

### 4.9. Extraction and Detection of AHL

The AHL production was obtained from bacterial culture supernatant using acidified ethyl acetate (0.1% glacial acetic acid in ethyl acetate) as previously described [41]. Cell-free supernatants were centrifugated at 12,000× *g* for 15 min and extracted with acidified ethyl acetate (0.1% glacial acetic acid in ethyl acetate) three times. The AHL extracts were concentrated by rotary evaporation. The AHL extracts were prepared for further assay.

The detection of AHL was determined by the CV026 biosensor and GC. The qualitative assay was performed following the methods of Joshi et al. [42] with some modifications. Briefly, agar plates were prepared by adding an overnight culture of the CV026 biosensor (1%) to the LB medium containing 0.4% agar supplemented with 100 µg/mL kanamycin. Then, 6-mm holes were punched into the agar, which were filled with the 50 µL AHL extracts. The test plates were incubated at 30 °C for 24 h. The AHL production was monitored by the size of diameter of the violacein. The experiments were replicated three times. For quantitative analysis, the C6-HSL was determined using a GC system (Acme 6100 GC, Korea YoungLin, Beijing, China) with a flame ionization detector (FID), both of which were controlled by a computer equipped with Autochro-2000 Chromatography Data System software (YoungLin, Korea). Sample (AHL extracts) injection was performed in split mode (20:1) into an HP-5MS capillary column (30 m × 25 mm × 0.25 µm). Hydrogen was used as the carrier gas at 1 mL/min. The GC injector temperature was set at 200 °C. The oven temperature program was optimized to hold at 100 °C for 1 min and then increased by 25 °C/min up to 280 °C. A standard curve was made with different concentrations of the C6-HSL standard and its corresponding peak areas. Then the C6-HSL concentration in *C. violaceum* was analyzed quantitatively.

### 4.10. Effect of *M. bracteata* EO on Signaling Molecules (C6-AHL)

The *C. violaceum* (1%, OD600, 0.9) was grown in 20 mL LB broth with or without EO (sub-MICs, 6% *v*/*v*) for 12 h at 30 °C and 150 rpm. Then, C6-HSL extracts were prepared for further assay by GC and CV026 biosensor.

In order to study the potential degradation of C6-HSL by *M. bracteata* EO, a known synthetic standard (C6-HSL) was added to 20 mL of LB broth untreated and treated with *M. bracteata* EO (sub-MICs, 6% *v*/*v*) and incubated for 0, 6, 12, and 24 h. The C6-HSL concentration was detected by GC.

The CV026 was grown in 20 mL of LB broth for 12 h with or without *M. bracteata* EO (sub-MICs, 6% *v*/*v*) supplemented with the exogenous C6-HSL. The C6-HSL extracts were prepared for further assay by GC.

### 4.11. Gene Expression Analysis

RT-qPCR was used to monitor the expression of QS genes of *C. violaceum*. The primers, which were used to amplify the *cviI*, *cviR* and other QS-regulated genes, are shown in Table S1. A total of 1% *C. violaceum* (OD600, 0.9) was used to incubate 20 mL of LB with or without a range of *M. bracteata* EO concentrations (sub-MICs, 6% *v*/*v*). Cultures were grown for 12 h, the cells were harvested by centrifugation (12,000× *g*, 2 min), and supernatants were discarded. Total RNA was extracted using an RNAprep Pure Cell/Bacteria Kit (Code No. DP430, TIANGEN, Beijing, China). The RNA was used for reverse-transcription using Transcript One-step gDNA Removal and cDNA Synthesis SuperMix (Transgen, Beijing, China). Quantitative RT-qPCR was performed using Real-time PCR Master Mix SYBR Green (Transgen, China) in a Bio-Rad C1000 Manager sequence detector system. The conditions were as follows: two steps of 30 s at 94 °C and 40 cycles of 94 °C for 5 s, 60 °C for 30 s. The calculated cycle threshold (CT) of each gene was normalized to the CT for *rpoD* amplified from the corresponding sample. The RT-qPCR was performed in a LightCycler 96. Fold changes in gene expression were calculated according the 2^−ΔΔCT^ method.

### 4.12. Statistical Analysis

All experiments were performed at least in triplicate and all data were analyzed by SPSS 19.0 software and presented as the mean values. Differences with *p* < 0.05 were considered statistically significant.

## 5. Conclusions

A total of 29 components were identified in *M. bracteata* EO, accounting for 96.49% of the total contents, among which methyleugenol displayed the largest proportion (90.46%), followed by methyl cinnamate (4.25%), and the relative content of other components was less than 1%.

The *M. bracteata* EO demonstrated significant inhibitory effects against seven pathogens, including *Dickeya dadantii* Onc5, *Staphylococcus aureus* ATCC25933, *Pseudomonas* spp., *Escherichia coli* ATCC25922, *Serratia marcescens* MG1, *Pseudomonas aeruginosa* PAO1, and *C. violaceum* ATCC31532. *S. aureus* ATCC25933 and *S. marcescens* MG1 had the highest sensitivity to *M. bracteata* EO, while *P. aeruginosa* PAO1 displayed the strongest resistance to *M. bracteata* EO.

The MIC of *M. bracteata* EO against *C. violaceum* was 10‰. The *M. bracteata* EO also interfered with the QS phenotype behaviors of *C. violaceum* without inhibiting its growth, primarily as follows: the violacein and biofilm were reduced with the maximum of inhibition rates of 85.47% and 75.56%, respectively. Biofilm formation and the swarming movement of *C. violaceum* were also inhibited after treatment with *M. bracteata* EO. The data showed that *M. bracteata* EO was capable of directly degrading C6-HSL and inhibiting the C6-HSL production. Furthermore, treatment with *M. bracteata* EO significantly repressed QS gene expression at sub-MIC concentrations.

Collectively, the anti-QS activity observed here lays a foundation for the development of *M. bracteata* EO into a new QS-inhibitor to prevent and control bacterial contamination. Further study of the interaction mechanism between the components of *M. bracteata* EO and bacteria should be analyzed in detail.

## Figures and Tables

**Table 1:** Chemical composition of volatile compounds in the *M. bracteata* EO.

NO.	Compounds Name	Molecular Formula	Molecular Weight	Relative Content	Retention Time/min	Retention Index
1	Methyleugenol	C_11_H_14_O_2_	178.23	90.46%	29.472	1399
2	Methyl trans-cinnamate	C_10_H_10_O_2_	162.1852	4.25%	28.864	1382
3	Estragole	C_10_H_12_O	148.2	0.32%	21.064	1194
4	alpha-Terpineol	C_10_H_18_O	154.25	0.23%	20.905	1190
5	3,7-Dimethyl-1,6-octadien-3-yl acetate 3,7 2-aminobenzoate	C_17_H_23_NO_2_	273.37	0.22%	16.42	1098
6	2,7-Dimethyl-2,6-octadien-1-ol	C_10_H_18_O	154.2493	0.18%	23.219	1240
7	Citronellol	C_10_H_20_O	156.27	0.14%	22.719	1229
8	2,2-Dimethoxybutane	C_6_H_14_O_2_	118.17	0.06%	3.626	-
9	3-Hexen-1-ol	C_6_H_12_O	100.16	0.05%	5.735	838
10	Z-Methyl geranate	C_11_H_18_O_2_	182.26	0.05%	26.75	1321
11	Myrcene	C_10_H_16_	136.23	0.05%	23.411	1244
12	Elemicin	C_12_H_16_O_3_	208.25	0.04%	33.566	1544
13	Citral	C_10_H_16_O	152.23	0.04%	24.591	1269
14	Terpinen-4-ol	C_10_H_18_O	154.25	0.03%	20.159	1175
15	Methyl propionate	C_4_H_8_O_2_	88.11	0.03%	2.154	-
16	Espatulenol	C_15_H_24_O	220.3505	0.03%	34.417	1575
17	(3aS,3bR,4S,7R,7aR)-7-methyl-3	C_15_H_24_	204.3511	0.03%	31.836	1481
18	Methyl 3,4,5-trimethoxybenzoate	C_11_H_14_O_5_	226.23	0.03%	36.955	1718
19	4-epi-cubedol	C_15_H_26_O	222	0.03%	36.201	1664
20	epi-a-Cadinol	C_15_H_26_O	222.3663	0.03%	36.368	1673
21	(R)-Lavandulyl acetate	C_12_H_20_O_2_	196	0.02%	21.881	1211
22	1,3,8-p-Menthatriene	C_10_H_14_	134.2182	0.02%	12.847	1100
23	2-Carene(7CI,8CI)	C_10_H_16_	136.234	0.02%	15.707	1083
24	Methyl butyrate	C_5_H_10_O_2_	102.13	0.02%	3.038	-
25	Decane	C_10_H_22_	142.28	0.02%	11.826	999
26	Dispiro[2.0.2.5]undecane, 8-methylene	C_12_H_18_	162.27132	0.02%	23.878	1254
27	Copaene(6CI)	C_15_H_24_	204.3511	0.02%	32.245	1495
28	2-(4-Methylphenyl)propan-2-ol	C_10_H_14_O	150.22	0.02%	20.484	1182
29	trans-α-Bergamotene	C_15_H_24_	204.35106	0.02%	23.382	1243
	Total			96.49%		

**Table 2:** Antibacterial activities of *M. bracteata* EO.

Bacterial Strains	Concentration/Antimicrobial Diameters (mm)						MIC
	80‰	40‰	20‰	10‰	Methanol	Kanamycin (250 µg/mL)	
*Dickeya dadantii* Onc5	11.89 ± 0.246 ^ah^	10.70 ± 0.291 ^abg^	10.03 ± 0.303 ^bcfgh^	9.00 ± 0.518 ^cfg^	6.00 ± 0.00	20.21 ± 0.11	10‰
*Staphylococcus aureus* ATCC25933	15.28 ± 1.083 ^ae^	13.05 ± 0.323 ^be^	10.98 ± 0.520 ^cef^	9.21 ± 0.078 ^def^	6.00 ± 0.00	26.01 ± 0.131	2.5‰
*Escherichia coli* ATCC25922	11.38 ± 0.286 ^ai^	10.15 ± 0.451 ^bgh^	9.325 ± 0.343 ^bh^	8.33 ± 0.354 ^cgh^	6.00 ± 0.00	18.78 ± 1.032	10‰
*Pseudomonas aeruginosa* PAO1	10.47 ± 0.186 ^aj^	9.82 ± 0.279 ^bh^	9.45 ± 0.236 ^ch^	8.15 ± 0.193 ^dh^	6.00 ± 0.00	17.23 ± 0.187	20‰
*Serratia marcescens* MG1	14.11 ± 0.789 ^af^	11.81 ± 0.363 ^bf^	10.57 ± 0.191 ^cefg^	9.87 ± 0.484 ^de^	6.00 ± 0.00	25.08 ± 1.31	2.5‰
*Pseudomonas* spp.	13.42 ± 0.715 ^ag^	12.49 ± 0.308 ^bef^	11.50 ± 0.236 ^ce^	9.69 ± 0.315 ^def^	6.00 ± 0.00	23.17 ± 0.33	5‰
*Chromobacterium violaceum* ATCC31532	11.55 ± 0.34 ^ai^	10.86 ± 0.49 ^ag^	9.80 ± 0.27 ^bgh^	8.03 ± 0.26 ^ch^	6.00 ± 0.00	21.61 ± 1.029	10‰

Note: Different letters (a–d) within the same row represent significant differences at the different concentrations (*p* < 0.05). Different letters (e–j) within the same line represent significant differences at the different concentrations (*p* < 0.05).

## Supplementary Materials

Supplementary materials can be found at https://www.mdpi.com/1422-0067/20/22/5696/s1.

## Abbreviations

QS	Quorum sensing
Anti-QS	Anti-quorum sensing
AHLs	*N*-acyl-homoserine lactones
C6-HSL	*N*-hexanoyl-l-homoserine lactone
MIC	Minimum inhibitory concentration
Sub-MIC	Sub-minimal inhibitory concentration
AI	Autoinducer
VF	virulence factor
EO	Essential oil
QSI	Quorum-sensing inhibitor

## References

[1] Iñiguez-Moreno M. (2018). Resistance of pathogenic and spoilage microorganisms to disinfectants in the presence of organic matter and their residual effect on stainless steel and polypropylene. J. Glob. Antimicrob. Resist..

[2] Savoia D. (2012). Plant-derived antimicrobial compounds: Alternatives to antibiotics. Future Microbiol..

[3] Wang L., Hu W., Deng J., Liu X., Zhou J., Li X. (2019). Antibacterial activity of *Litsea cubeba* essential oil and its mechanism against *Botrytis cinerea*. RSC Adv..

[4] Chang C.Y., Krishnan T., Wang H., Chen Y., Yin W.F., Chong Y.M., Tan L.Y., Chong T.M., Chan K.G. (2014). Non-antibiotic quorum sensing inhibitors acting against N-acyl homoserine lactone synthase as druggable target. Sci. Rep..

[5] Zhou J.W., Luo H.Z., Jiang H., Jian T.K., Chen Z.Q., Jia A.Q. (2018). Hordenine, a novel quorum sensing inhibitor and anti-biofilm agent against *Pseudomonas aeruginosa*. J. Agric. Food Chem..

[6] Haque S., Ahmad F., Dar S.A., Jawed A., Mandal R.K., Wahid M., Lohani M., Khan S., Singh V., Akhter N. (2018). Developments in strategies for Quorum Sensing virulence factor inhibition to combat drug resistant bacteria. Microb. Pathog..

[7] Kerekes E.B., Deák É., Takó M., Tserennadmid R., Petkovits T., Vágvölgyi C., Krisch J. (2013). Anti-biofilm forming and anti-quorum sensing activity of selected essential oils and their main components on food-related micro-organisms. J. Appl. Microbiol..

[8] Jamuna B.A., Vittal R.R. (2014). Quorum Sensing Inhibitory and Anti-Biofilm Activity of Essential Oils and Their in vivo Efficacy in Food Systems. Food Biotechnol..

[9] Kothari V., Sharma S., Padia D. (2017). Recent research advances on *Chromobacterium violaceum*. Asian Pac. J. Trop. Med..

[10] Durán N., Justo G.Z., Durán M., Brocchi M., Cordi L., Tasic L., Castro G.R., Nakazato G. (2016). Advances in *Chromobacterium violaceum* and properties of violacein-Its main secondary metabolite: A review. Biotechnol. Adv..

[11] Eris R., Ulusoy S. (2013). Rose, clove, chamomile essential oils and pine turpentine inhibit quorum sensing in *Chromobacterium violaceum* and *Pseudomonas aeruginosa*. J. Essent. Oil Bear. Plants.

[12] Asghar A., Butt M.S., Shahid M., Huang Q. (2017). Evaluating the antimicrobial potential of green cardamom essential oil focusing on quorum sensing inhibition of *Chromobacterium violaceum*. J. Food Sci. Technol..

[13] Li C., Liu H., Zhao L., Zhang W., Qiu S., Yang X., Tan H. (2017). Antibacterial neolignans from the leaves of *Melaleuca bracteata*. Fitoterapia.

[14] Hou W., Zhang W., Chen G., Luo Y. (2016). Optimization of Extraction Conditions for Maximal Phenolic, Flavonoid and Antioxidant Activity from *Melaleuca bracteata* Leaves Using the Response Surface Methodology. PLoS ONE.

[15] Adesanwo K.J., Shode F.O., Aiyelaagbe O.O., Rabiu O.O., Oyede R.T., Oluwole F.S. (2009). Antisecretory and antiulcerogenic activities of the stem bark extract of *Melaleuca bracteata* and isolation of principles. J. Med. Plants Res..

[16] Siddique S., Parveen Z., Mazhar S. (2017). Chemical composition, antibacterial and antioxidant activities of essential oils from leaves of three Melaleuca species of Pakistani flora. Arab. J. Chem..

[17] Li Y., Ye Z., Wang W., Yang C., Liu J., Zhou L., Shen Y., Wang Z., Chen J., Wu S. (2018). Composition Analysis of Essential Oil from *Melaleuca bracteata* Leaves Using Ultrasound-assisted Extraction and its Antioxidative and Antimicrobial Activities. BioResources.

[18] Durán M., Faljoni-Alario A., Durán N. (2010). *Chromobacterium violaceum* and its important metabolites--review. Folia Microbiol..

[19] Husain F.M., Ahmad I., Althubiani A.S., Abulreesh H.H., Alhazza I.M., Aqil F. (2017). leaves Extracts of Mangifera indicaL. Inhibit Quorum Sensing—Regulated Production of Virulence Factors and Biofilm in Test Bacteria. Front. Microbiol..

[20] Verstraeten N., Braeken K., Debkumari B., Fauvart M., Fransaer J., Vermant J., Michiels J. (2008). Living on a surface: Swarming and biofilm formation. Trends Microbiol..

[21] Ghosh R., Tiwary B.K., Kumar A., Chakraborty R. (2014). Guava leaves Extract Inhibits Quorum-Sensing and *Chromobacterium violaceum* Induced Lysis of Human Hepatoma Cells: Whole Transcriptome Analysis Reveals Differential Gene Expression. PLoS ONE.

[22] Brito C.F., Carvalho C.B., Santos F., Gazzinelli R.T., Oliveira S.C., Azevedo V., Teixeira S.M. (2004). *Chromobacterium violaceum* genome: Molecular mechanisms associated with pathogenicity. Genet. Mol. Res..

[23] Bacha K., Tariku Y., Gebreyesus F., Zerihun S., Mohammed A., Weiland-Bräuer N., Schmitz R.A., Mulat M. (2016). Antimicrobial and anti-Quorum Sensing activities of selected medicinal plants of Ethiopia: Implication for development of potent antimicrobial agents. BMC Microbiol..

[24] Truchado P., López-Gálvez F., Gil M.I., Tomás-Barberán F.A., Allende A. (2009). Quorum sensing inhibitory and antimicrobial activities of honeys and the relationship with individual phenolics. Food Chem..

[25] Taganna J.C., Quanico J.P., Perono R.M.G., Amor E.C., Rivera W.L. (2011). Tannin-rich fraction from *Terminalia catappa* inhibits quorum sensing (QS) in *Chromobacterium violaceum* and the QS-controlled biofilm maturation and LasA staphylolytic activity in *Pseudomonas aeruginosa*. J. Ethnopharmacol..

[26] Zhu H., He C.C., Chu Q.H. (2011). Inhibition of quorum sensing in *Chromobacterium violaceum* by pigments extracted from *Auricularia auricular*. Lett. Appl. Microbiol..

[27] Packiavathy I.A.S.V., Priya S., Pandian S.K., Ravi A.V. (2014). Inhibition of biofilm development of uropathogens by curcumin—An anti-quorum sensing agent from *Curcuma longa*. Food Chem..

[28] Adhavan P., Kaur G., Princy A., Murugan R. (2017). Essential oil nanoemulsions of wild patchouli attenuate multi-drug resistant gram-positive, gram-negative and *Candida albicans*. Ind. Crops Prod..

[29] Packiavathy I.A.S.V., Agilandeswari P., Musthafa K.S., Pandian S.K., Ravi A.V. (2012). Antibiofilm and quorum sensing inhibitory potential of *Cuminum cyminum* and its secondary metabolite methyl eugenol against Gram negative bacterial pathogens. Food Res. Int..

[30] Husain F.M., Ahmad I., Khan M.S., Ahmad E., Tahseen Q., Khan M.S., Alshabib N.A. (2015). Sub-MICs of Mentha piperita essential oil and menthol inhibits AHL mediated quorum sensing and biofilm of Gram-negative bacteria. Front. Microbiol..

[31] Stauff D.L., Bassler B.L. (2011). Quorum sensing in *Chromobacterium violaceum*: DNA recognition and gene regulation by the *cviR* receptor. J. Bacteriol..

[32] Morten H., Michael G. (2003). Pharmacological inhibition of quorum sensing for the treatment of chronic bacterial infections. J. Clin. Investig..

[33] Swem L.R., Swem D.L., O’Loughlin C.T., Gatmaitan R., Zhao B., Ulrich S.M., Bassler B.L. (2009). A Quorum-Sensing Antagonist Targets Both Membrane-Bound and Cytoplasmic Receptors and Controls Bacterial Pathogenicity. Mol. Cell.

[34] Goswami P., Verma S.K., Chauhan A., Venkatesha K., Verma R.S., Singh V.R., Darokar M.P., Chanotiya C.S., Padalia R.C. (2017). Chemical Composition and Antibacterial Activity of *Melaleuca bracteata* Essential Oil from India: A Natural Source of Methyl Eugenol. Nat. Prod. Commun..

[35] Lucero M., Estell R., Tellez M., Fredrickson E. (2010). A retention index calculator simplifies identification of plant volatile organic compounds. Phytochem. Anal..

[36] Dool H.V.D., Kratz P.D. (1963). A generalization of the retention index system including linear temperature programmed gas—Liquid partition chromatography. J. Chromatogr. A.

[37] Andotra S., Kalgotra N., Pandey S.K. (2014). Syntheses, Characterization, Thermal, and Antimicrobial Studies of Lanthanum(III) Tolyl/Benzyldithiocarbonates. Bioinorg. Chem. Appl..

[38] Vasavi H.S., Arun A.B., Rekha P.D. (2015). Anti-quorum sensing potential of Adenanthera pavonina. Pharmacogn. Res..

[39] Zhang Y., Kong J., Huang F., Xie Y., Guo Y., Cheng Y., Qian H., Yao W. (2018). Hexanal as a QS inhibitor of extracellular enzyme activity of Erwinia carotovora and *Pseudomonas* fluorescens and its application in vegetables. Food Chem..

[40] O’Toole G.A. (2011). Microtiter dish biofilm formation assay. J. Vis. Exp. Jove.

[41] Blosser R.S., Gray K.M. (2000). Extraction of violacein from *Chromobacterium violaceum* provides a new quantitative bioassay for N-acyl homoserine lactone autoinducers. J. Microbiol. Methods.

[42] Joshi J.R., Khazanov N., Senderowitz H., Burdman S., Lipsky A., Yedidia I. (2016). Plant phenolic volatiles inhibit quorum sensing in *pectobacteria* and reduce their virulence by potential binding to ExpI and ExpR proteins. Sci. Rep..

