# Supplementary material for: Antibacterial Activity and Anti-Quorum Sensing Mediated Phenotype in Response to Essential Oil from Melaleuca bracteata Leaves
Source: Int J Mol Sci. 2019 Nov 14;20(22):5696. doi: 10.3390/ijms20225696 (PMC6887945; doi:10.3390/ijms20225696)
Supplement: Supplementary file 1 [file ijms-20-05696-s001.pdf]

Table S1. Primers for fluorescence real-time quantitative PCR.

Gene	Forward (5′–3′)	Reverse (5′–3′)
cviI	GAAACCGTCCTCGCATAAGG	ACAAGGTGGACTGGTACTGG
cviR	CCCAGCAATATGCCGCTATC	CATTGAGCTTGCGGATCACA
vioA	AAGAGCATGGCAAGGAATC	CTGGTTGGCGTCGTTATC
vioB	CTGGGCGTAATTGGGAATGG	CAAATACCTGGCCCATGTCG
vioC	GAACAAGTACGCCAACCT	GGAAGAAAGTCTGCTGGAA
vioD	GCCGCAACAAGTACATCT	GAAGGTGCTCATCGTGTC
vioE	ATAGGCCACCTTCTGCTTCC	GGCTACTGCTGGTTCGACTA
hmsH	CGCCGTATGTCTTCAGTT	CGCAGCCTATCGTAGATG
hmsF	GGCTGCTGATTCTCTGTTA	GTATAGACGCTGCGGTAG
hmsN	AAACCACACGCACCAGAAC	TGCATGAGCATGAAGACGAC
lasA	AGCCAGCCTTACGATTCCAT	GAGGAATAGCCGTTGTCGTG
lasB	AGAACGCCTTGTTGTACACG	GCAAGAACGACTTCCTGGTC
hcnA	CTCCGTATTCAGACGGTTGC	ACTGCTGCCTGGTCAAGATA
hcnB	AATCTCTTCAGTCTGTTGGG	GATGTGCGTCGGCTATTG
hcnC	ATAGGTGATGGCGGTGTC	GTGCTGTCGGAGAAGATG
PilE1	CCTGCTGGAACTGGTGAT	GCTGGCGTAGTTGTTGTT
pilE2	GAGCCGGTATAGGTGCAGTA	GTGAGCAGCATCAACGTTCT
pilE3	TACGCTGGTCGAGTTGATGA	GCGAATAGCACTGCTCCATC
rpoD (actin)	TCGGACATCAGCAAGGTT	GTGAAGGACAGCCAACAG

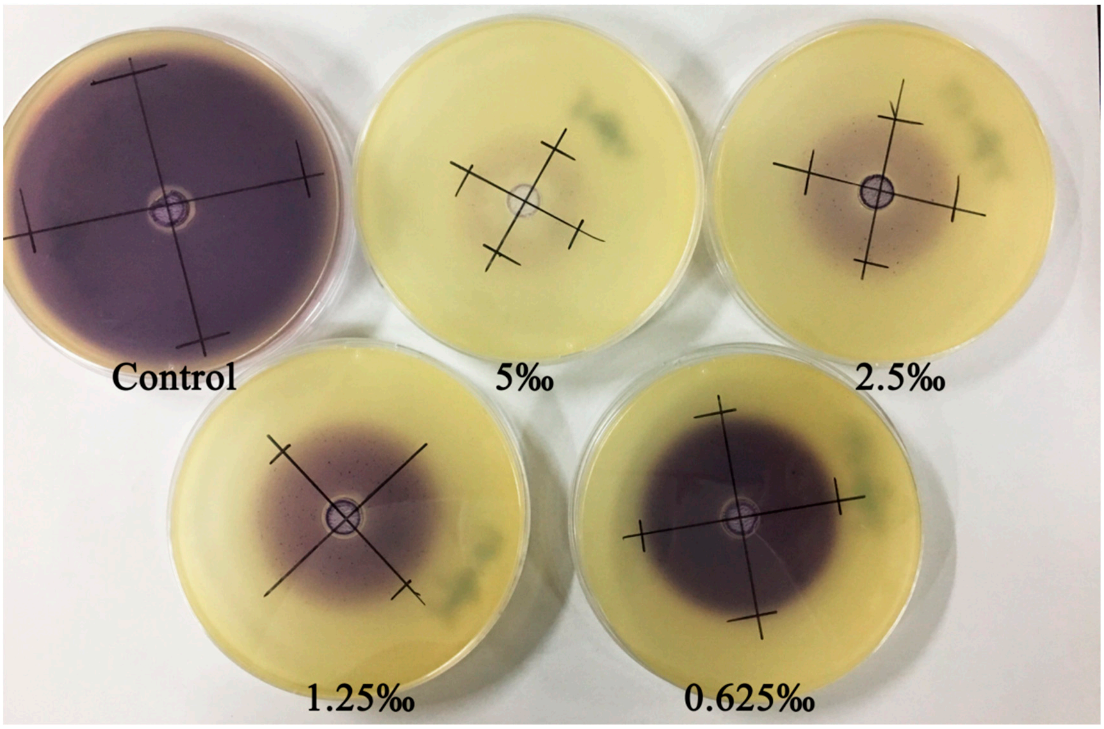

Figure S1. Induction of purple color production in CV026 by supernatants of *C. violaceum* culture grown in LB, untreated and treated with different concentrations of *M. bracteata* EO. Control (untreated with EO), 5‰, 2.5‰, 1.25‰, and 0.625‰.

A

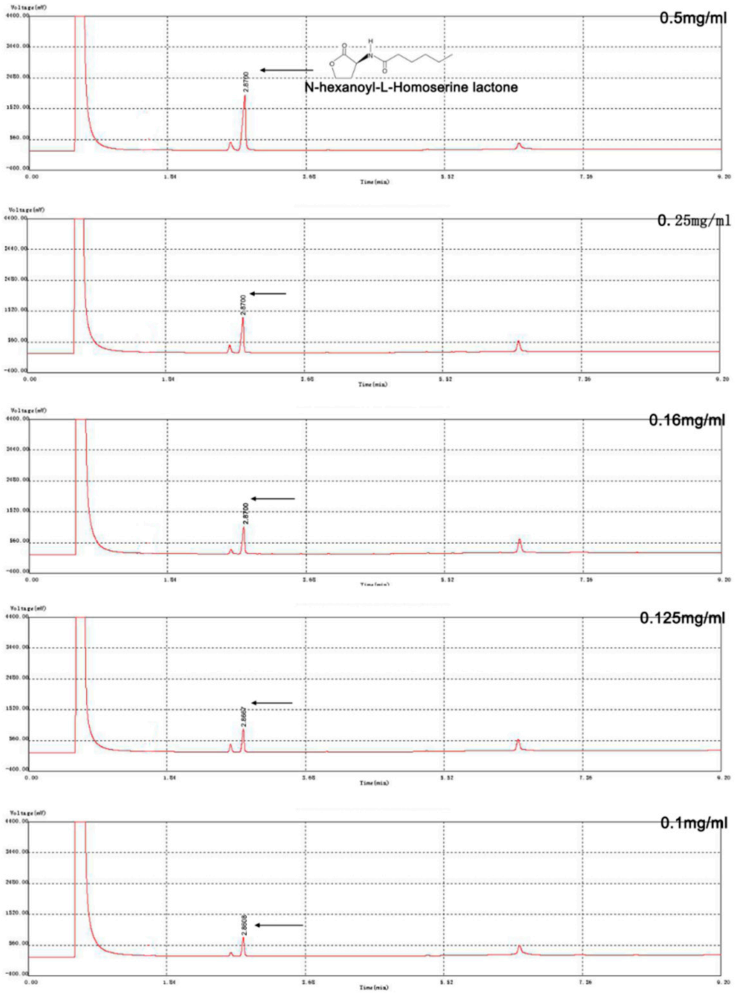

B

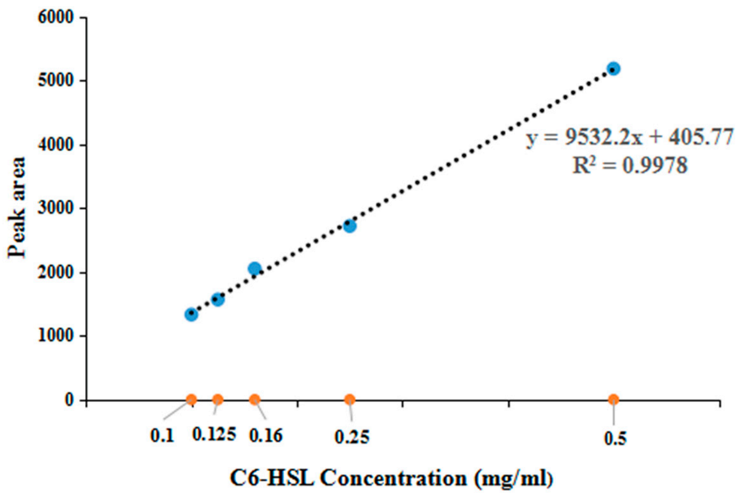

Figure S2. GC determination of the standard curve of C6-HSL (**A**). The standard curve of C6-HSL (**B**).

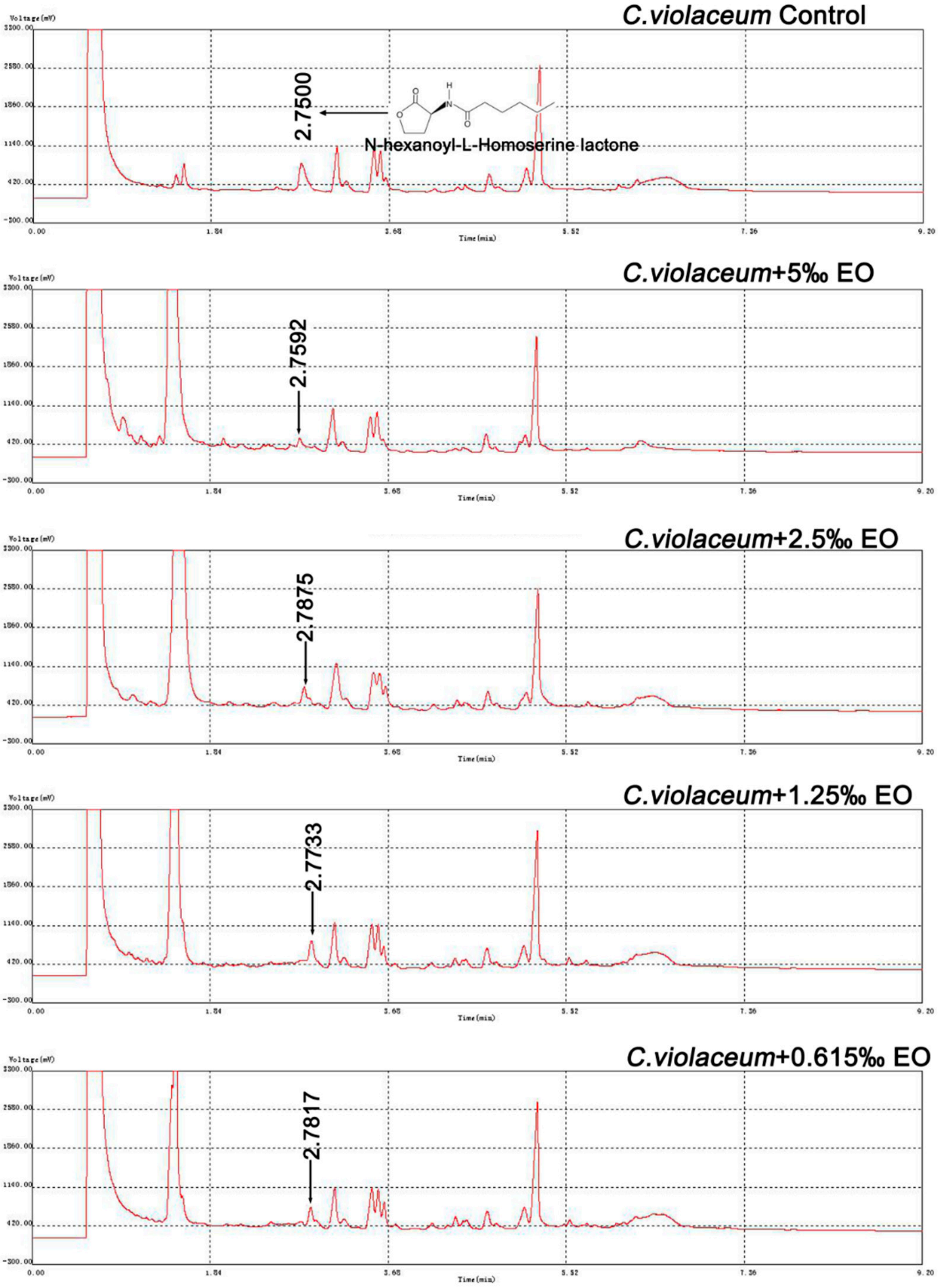

Figure S3. GC analysis of the effect of *M. bracteata* EO at different concentration (5‰, 2.5‰, 1.25‰, and 0.625‰) on C6-HSL production of *C. violaceum.*

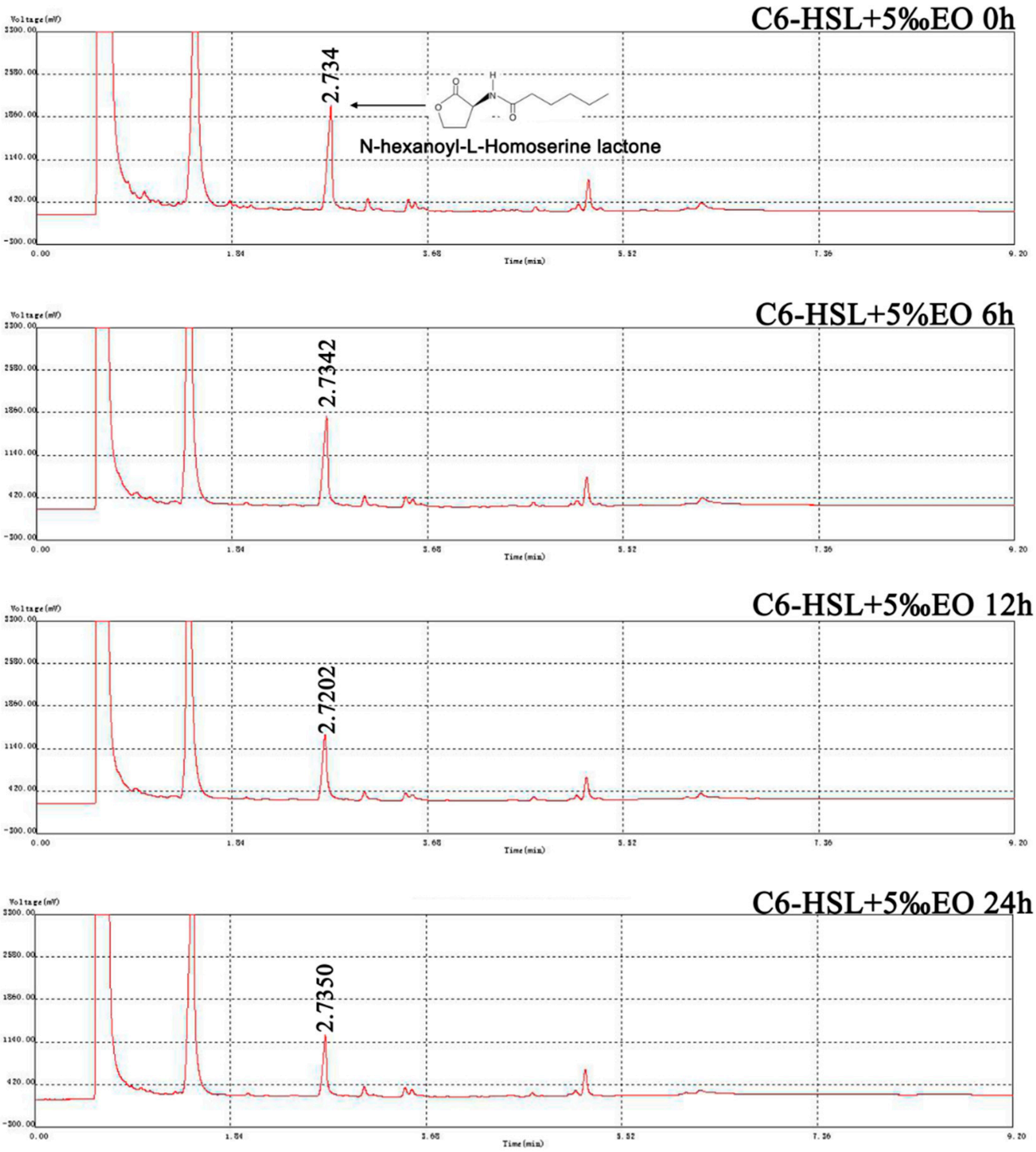

Figure S4. GC analysis of the effect of *M. bracteata* EO (5‰) on C6-HSL treated for 0, 6, 12, and 24 h.

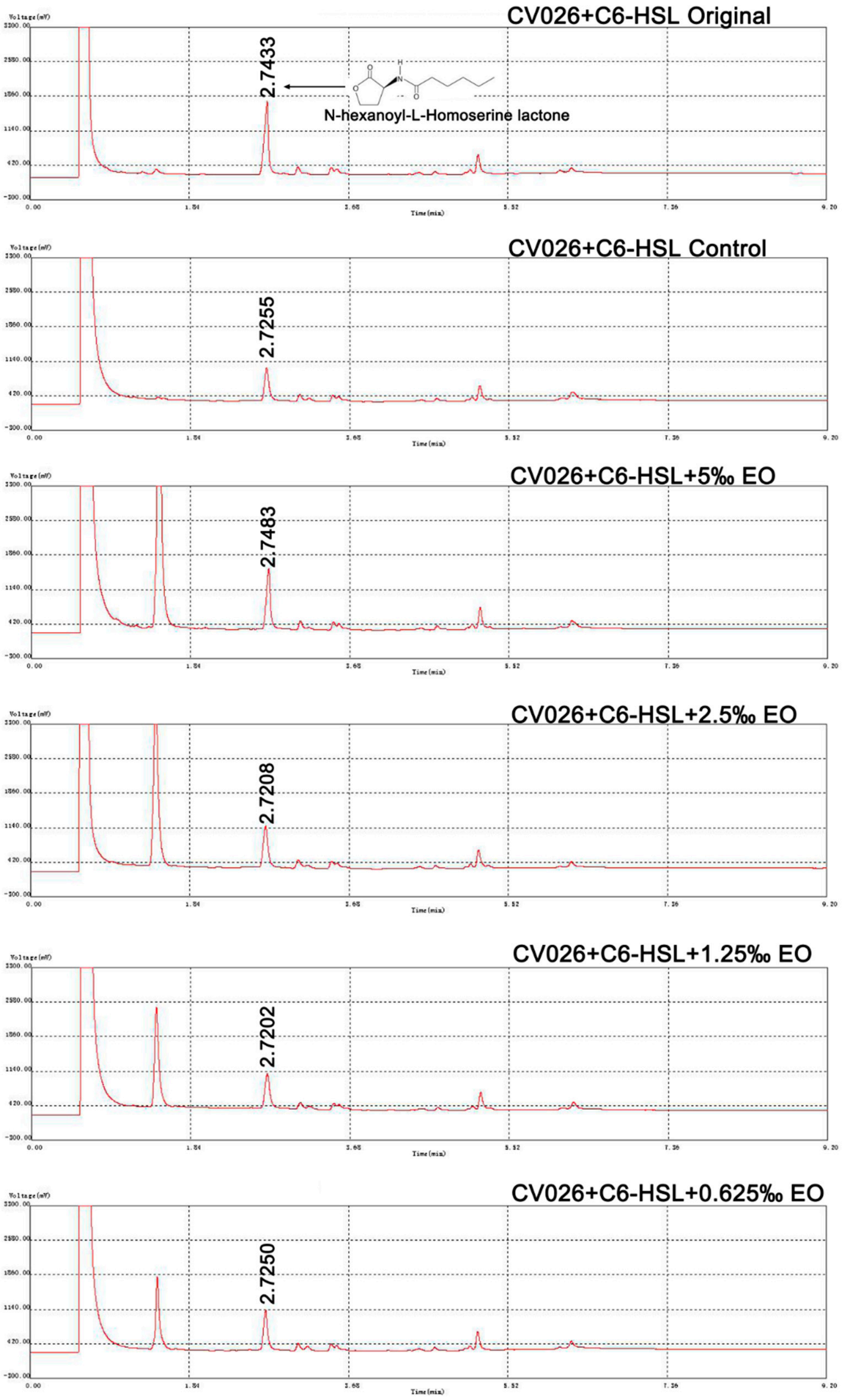

Figure S5. GC analysis of the effect of *M. bracteata* EO at different concentrations (5‰, 2.5‰, 1.25‰m and 0.625‰) on C6-HSL of *C. violaceum* CV026.

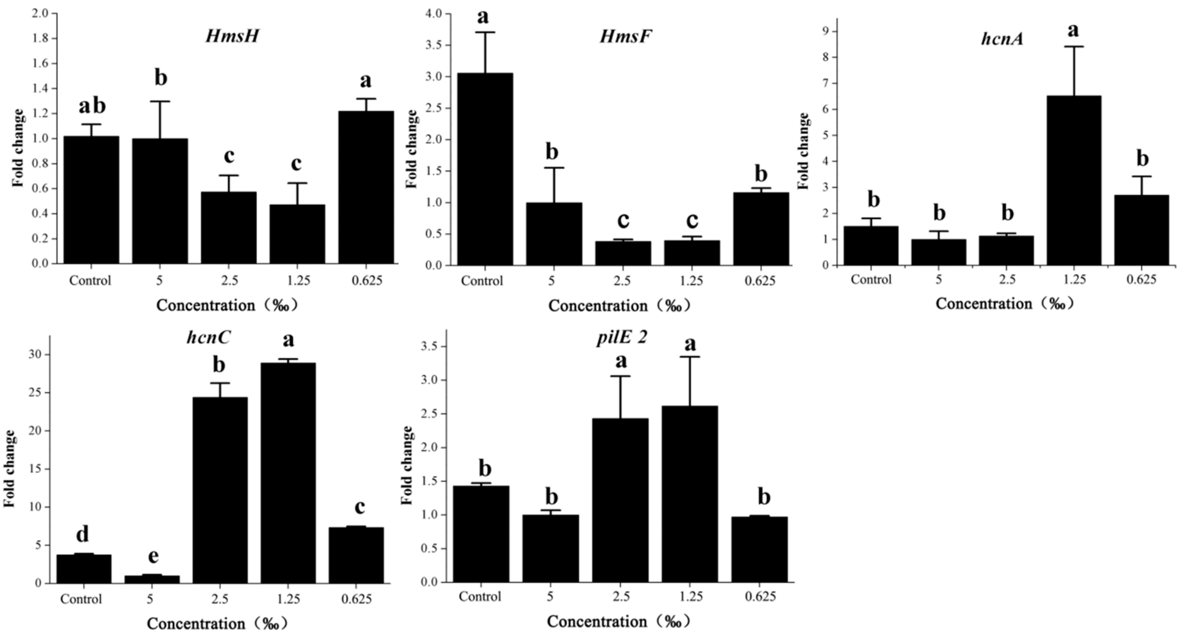

Figure S6. Effect of *M. bracteata* EO on the expression of virulence genes related to QS . hmsH, hmsF, hcnA, hcnC and pilE2 were detected in response to *M. bracteata* EO treatment. Expression of the house-keeping gene *rpoD* was used as the internal control for each sample. The *M. bracteata* EO treatment concentrations were: 5‰, 2.5‰, 1.25‰, and 0.625‰. Control was untreated.

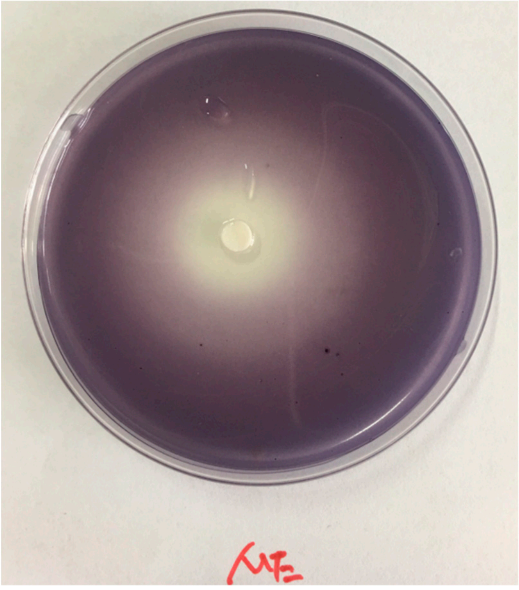

Figure S7. QSI effect of methyleugenol (ME) on biosensor CV026.
